# Pharmacokinetics of Transdermal Flunixin Meglumine Following a Single Dose in Marine Toads (*Rhinella marina*)

**DOI:** 10.1155/2020/8863537

**Published:** 2020-08-27

**Authors:** Gregory Scott, Meghan M. Louis, Julie A. Balko, Claire M. Bublitz, Brigid V. Troan, Ronald E. Baynes, Dustin Smith, Larry J. Minter

**Affiliations:** ^1^Department of Clinical Sciences, College of Veterinary Medicine, North Carolina State University, 1060 William Moore Drive, Raleigh, NC 27607, USA; ^2^Center for Marine Sciences and Technology, North Carolina State University, 303 College Circle, Morehead City, NC 28557, USA; ^3^Hanes Veterinary Medical Center, North Carolina Zoo, 4401 Zoo Parkway, Asheboro, NC 27205, USA; ^4^Environmental Medicine Consortium, North Carolina State University, 1060 William Moore Drive, Raleigh, NC 27607, USA; ^5^Department of Molecular Biomedical Sciences, College of Veterinary Medicine, North Carolina State University, 1060 William Moore Drive, Raleigh, NC 27607, USA; ^6^Department of Population Health and Pathobiology, College of Veterinary Medicine, North Carolina State University, 1060 William Moore Drive, Raleigh, NC 27607, USA

## Abstract

Transdermal administration is an important method of pharmacologic drug therapy in amphibians, made possible by their unique skin physiology and permeability. Despite this, there are relatively few studies that investigate transdermal pharmacokinetics in amphibians. The objective of this study was to investigate the pharmacokinetics of transdermal flunixin meglumine applied topically to marine toads (*Rhinella marina*). Twenty-one adult marine toads were administered flunixin meglumine (3.3 mg/kg) topically on their dorsum and randomized (*n* = 7/group) to blood collection at two timepoints from the following: 1, 2, 4, 8, 12, and 24 hours (h), using a sparse sampling protocol. Plasma was analyzed by ultra-performance liquid chromatography-tandem mass spectrometry. Samples were analyzed individually and reported as a mean of the samples at each timepoint. The mean peak plasma concentration was 6.31 *µ*g/ml, area under the curve was 29.37 *μ*g-h/mL, and elimination half-life was 2.79 h. No adverse effects were noted in any animals. A subset of 12 animals were euthanized at serial timepoints and necropsied. Histopathology of skin and major organs revealed one minimal superficial lesion in a single toad potentially attributable to flunixin meglumine administration; otherwise, no other treatment-related lesions were observed in 11 of 12 toads. A single topical dose of transdermal flunixin meglumine was rapidly absorbed in marine toads in the current study, and peak plasma concentrations exceeded therapeutic ranges established in cattle with no significant pathologic findings.

## 1. Introduction

There is growing evidence to support the use of nonsteroidal anti-inflammatory drugs (NSAIDs) for analgesia in amphibians, including flunixin meglumine, meloxicam, and ketoprofen among others [1–6]. NSAIDs act by antagonizing cyclooxygenase (COX) enzymes that activate the inflammatory cascade by converting arachidonic acid into prostaglandins [7]. The same mechanism is supported in an amphibian model using meloxicam. In North American bullfrogs (*Lithobates catesbeianus*) that underwent mild surgical trauma, an intramuscular injection of meloxicam suppressed the production of prostaglandin E2 (PGE2) [4]. Studies have also shown that NSAIDs provide clinically effective analgesia in amphibians. Indomethacin and ketorolac administered by subcutaneous injection produced mild analgesic effects in northern leopard frogs (*Lithobates pipiens*) [2]. Compared to saline controls, treated frogs had an increased nociceptive threshold in response to an acetic acid test, where increasing concentrations of acetic acid are applied topically as a noxious stimulus until the animal exhibits a response. Acetic acid tests have also shown that flunixin meglumine administered by intracoelomic injection provides analgesia in leopard frogs [1]. Analgesia has been demonstrated in African clawed frogs (*Xenopus laevis*) treated with subcutaneous flunixin meglumine, assessed by both an acetic acid test and the Hargraves tests, the latter of which exposes the animal's limb to a thermal noxious stimulus, and latency withdrawal times are recorded [3, 6].

Despite the established therapeutic benefit of NSAIDs, the small body size and sensitive skin of amphibians creates challenges for safe handling and delivery of injectable or oral medications. The permeability of amphibian skin, however, offers a unique opportunity for transdermal drug absorption following topical administration. The skin is a major interface of gas, water, and electrolyte exchange for many amphibians, and any drug applied to the skin has potential for systemic uptake [8, 9]. For amphibians, recommendations are available for transdermal use of multiple classes of therapeutics including antibiotics, antifungals, antiparasitics, anesthetics, analgesics, and nutritional supplements [9]. However, absorption of topically applied drugs can be variable [5]. While there are reports of their usage, few drugs have pharmacokinetic studies to support transdermal absorption in amphibians [10–13]. Only one study has evaluated plasma concentrations following topical application of the NSAIDs ketoprofen and meloxicam; however, neither was formulated for transdermal delivery [5].

In 2017, a novel transdermal formulation of flunixin meglumine was approved by the US Food and Drug Administration for use in cattle for the control of pain and fever. Studies in cattle have shown that this formulation is rapidly absorbed and effective for reducing lameness, cortisol, and PGE2 in inflammatory exudates [14–17]. Given the usefulness of topical therapy in amphibians, the targeted nature of this drug for topical use, and the effectiveness of this formulation in at least one species, investigation of transdermal flunixin meglumine for use in amphibians is warranted.

The objective of this study was to examine the pharmacokinetics of this newly available transdermal flunixin meglumine formulation in healthy marine toads (*Rhinella marina*) following a single topical dose. Our hypothesis was that flunixin meglumine would be rapidly absorbed and reach peak plasma concentrations consistent with those recommended for therapy in cattle.

## 2. Materials and Methods

This study was approved by the Institutional Care and Use Committee of North Carolina State University, IACUC ID# 19-614. Twenty-one adult marine toads of undetermined sex and a median (range) weight of 117 g (84.5–206.5 g) were enrolled in the study. The toads were wild-caught as part of a routine invasive species management program from a free-ranging feral population on the grounds at a zoological institution in Miami, Florida, USA. A subset of animals was approved by the Zoo Miami Research Committee to be transferred to the North Carolina Zoo in Asheboro, North Carolina (NC Zoo), for use in this study. Prior to transfer, all animals were individually identified with subcutaneous passive integration transponders. Upon arrival at the NC Zoo, the animals were divided and housed in four separate tubs (178.8 × 81.28 × 35.56 cm) with access to hide boxes and a shallow pool of reconstituted reverse osmosis water. The tanks were cleaned daily and disinfected weekly. The animals were kept at room temperature without supplemental heating or cooling. Humidity was not measured. The light cycle was maintained according to the working hours of the animal husbandry staff (8 : 00 am–5 : 00 pm). Animals were group fed approximately 10 crickets (gut-loaded and dusted with calcium powder) per toad for five consecutive days per week. All animals were allowed a 6-day acclimation period and determined to be healthy based on a physical exam by a veterinarian prior to the study. Animals did not receive any other medical treatments during the study.

**Figure 1:**
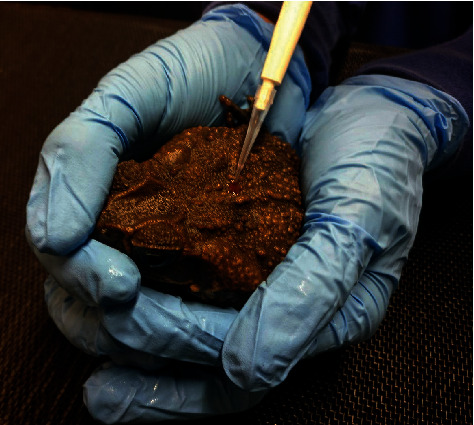
Image of transdermal flunixin meglumine administration location on the central dorsum of a marine toad using a micropipette.

On the study day, a target dose of 3.3 mg/kg transdermal flunixin meglumine (Banamine Transdermal, Merck Animal Health, Madison, New Jersey 07940, USA) was administered topically to each toad on the midpoint of the dorsum (Figure 1). The dose was chosen based on the treatment recommendation for cattle [14, 16]. Higher doses (25–50 mg/kg) previously reported in frogs were avoided due to renal pathology and mortalities associated with those treatments [3, 6]. Each animal was individually removed and weighed just prior to being treated. The animals were briefly manually restrained to ensure consistent drug placement. Due to small dose volumes, each dose was measured and administered with a 5–20 *μ*L micropipette (Fisher Scientific, Waltham, Massachusetts 02451, USA). All toads were placed in a separate tub following treatment and restricted from having access to water for a minimum of 4 hours following drug administration. If samples beyond 4 hours were required, toads were returned to their original tub and given access to shallow water below the level of the dorsum for the remainder of the study.

The toads were randomly divided [18] into three groups (*n* = 7/group) for timing of blood collection. Each group had blood collected at two different timepoints from the following: 1, 2, 4, 8, 12, and 24 h after flunixin meglumine administration. This approach represents a sampling method according to a population pharmacokinetic matrix using sparse sampling technique to minimize handling required for multiple sample collections on each animal [19]. Each toad was manually restrained for blood collection. Blood was collected from the popliteal sinus using a 27-gauge needle and syringe and immediately transferred to a lithium heparin blood tube. All blood samples were centrifuged, and plasma was separated into cryovials and stored at −80°C within 1 hour (h) of collection. Total blood sampling did not exceed 1% body weight for each animal for the duration of the study. Once all samples were collected, frozen samples were transported to North Carolina State University College of Veterinary Medicine for flunixin meglumine quantification.

Plasma was analyzed by liquid chromatography (UPLC/MS/MS) utilizing a BEH Phenyl 1.7 *μ*m 2.1 × 100 mm column. Drug standards (Sigma Aldrich, St. Louis, Missouri 63103, USA) for flunixin meglumine were >99% purity. Corrections were made for the meglumine salt in flunixin prior to standards being developed. The UPLC/MS/MS method was validated using blank plasma from toads. For extraction, plasma samples were thawed and processed using a solid phase extraction (SPE), and 50 *μ*L of each sample was combined with 250 *μ*L of 4% phosphoric acid in ultrapure water. Samples were vortexed and then transferred to an Oasis Prime HLB 1 cc Cartridge (Waters Corp., Milford, Massachusetts 01757, USA). The samples were processed with the nitrogen positive pressure manifold (Biotage, Charlotte, North Carolina 28269, USA). Each sample was washed with 1 mL of 5 : 95 methanol : ultrapure water and eluted with 0.5 mL of 60 : 40 acetonitrile : ultrapure water +0.1% formic acid. The samples were then filtered through 0.2 *μ*m PTFE Whatman Mini-UniPrep syringe filter devices and analyzed by UPLC/MS/MS. The injection volume for all samples was 5 *μ*L. Validation standards were prepared over a linear range and were used to construct calibration curves. The standard curve concentrations ranged from 0.5 ng/mL to 10,000 ng/mL. The limit of quantification (LOQ) was 1 ppb, and the limit of detection (LOD) was 0.5 ppb, which was the lowest concentration on the calibration curve. The column temperature was 35°C, the sample temperature was ambient, and the run time was 5 minutes.

Pharmacokinetic analysis of drug concentration versus time profiles was performed with Phoenix WinNonlin software (version 8.0; Certara, Princeton, NJ, 08540 USA). A noncompartmental analysis of sparse samples was used to derive the slope of the terminal phase (*λ*_z_; h) and the half-life (*t*_1/2_; h). The area under the plasma concentration–time curve from time zero to infinity (AUC_0⟶∞_; h × *μ*g/mL) was calculated by the linear trapezoidal rule. The volume of distribution (per fraction absorbed) (Vd/F; ml/kg) and clearance per fraction absorbed (Cl/F; ml/h/kg) were also determined, and values for maximum concentration (*C*_max_; *µ*g/mL) and time to maximum concentration (*T*_max_; h) were taken directly from the data. Population pharmacokinetic analysis was attempted using Phoenix NLME. The NLME attempts yielded similar parameter estimates to the noncompartmental analysis, but due to limited data and high shrinkage, the variability would not have been fully represented, so it does not provide any more information than noncompartmental analysis.

Three toads were euthanized at 48 h, and nine toads were euthanized 8-9 days after flunixin meglumine treatment; complete necropsies were performed, and tissues were fixed in 10% neutral buffered formalin. Tissues were processed routinely for evaluation by light microscopy by a board-certified veterinary pathologist. For the first three toads, two sections of skin each from the treated and untreated areas as well as liver and kidney were examined. In the remaining 9 toads, two sections of skin each from the treated and untreated areas and drink patch, along with brain, lung, heart, liver, kidney, spleen, gastrointestinal tract, and gonad, were evaluated.

## 3. Results

**Figure 2:**
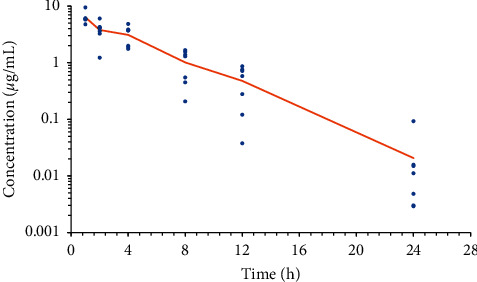
Semilogarithmic plot of all plasma flunixin meglumine concentrations following topical administration (3.3 mg/kg) in marine toads (*n* = 21). The line plot represents the average values at each timepoint.

Transdermal flunixin meglumine was administered successfully to all 21 toads. No animals displayed any adverse responses or effects during the study. All blood samples were collected without difficulty with the exception of one toad at the 1 h timepoint at which blood could not be collected. A scatter plot of the plasma flunixin meglumine concentrations of each toad with a line plot of the mean values over time is presented in Figure 2. The pharmacokinetic parameters are presented in Table 1.

On necropsy, no gross lesions attributable to flunixin meglumine treatment were appreciated in any of the toads. Histologically, one toad had an approximately 1000 *µ*m diameter, well-demarcated section of treated skin showing superficial epidermal necrosis with clefting through the stratum spinosum to above the stratum basale, dissociation of cells within the remaining epidermis, and a mild lymphoplasmacytic junctional infiltrate. This was interpreted as minimal, focal, superficial epidermal necrosis, consistent with topical injury. However, this finding could not be confidently attributed to the topical flunixin meglumine as other possibilities such as an insect bite or direct injury from the pipette tip could not be ruled out. No further histologic lesions potentially attributable to treatment were observed in the skin or organs of any of the other 11 toads examined.

## 4. Discussion

Data from the current study indicate that topically applied transdermal flunixin meglumine at 3.3 mg/kg is rapidly absorbed in marine toads. The mean maximum plasma concentration (*C*_max_) in marine toads is more than 5-fold that of dairy cattle also administered 3.3 mg/kg [14, 16]. A follow-up study determined that in cattle with induced lameness, transdermal flunixin meglumine at 3.3 mg/kg improved lameness scores, reduced plasma cortisol concentration, reduced coronary band temperature, and increased pain tolerance threshold compared to untreated animals [17]. In goats, transdermal flunixin meglumine at 3.3 mg/kg resulted in a *C*_max_ of 0.134 *μ*g/mL and a 50% reduction from baseline of plasma PGE2 concentration [20]. As *C*_max_ in the current study was nearly 50 times greater (6.31 *μ*g/mL), one could hypothesize that this dosing in marine toads would be associated with similar indicators of effective analgesia.

Time to reach maximum plasma concentration (*t*_max_) in toads was 1 h, which is faster than dairy cattle, goats, and pigs [14, 16, 20, 21]. Furthermore, it should be noted that the current study failed to observe the absorption phase as the first sampling time (1 h) yielded the highest mean concentration. Thus, plasma concentrations likely reached a higher *C*_max_ at a time point sooner than 1 h, but it was not detected by this sampling protocol. With the rapid plasma uptake, it is likely that resulting analgesic effects occur quickly as well. Based on these results, future studies into the analgesic effects of transdermal flunixin in amphibians are warranted as it may provide rapid clinical analgesia without the need for injection.

The plasma elimination half-life (*t*_1/2_) in toads (2.79 h) was relatively short compared to 6- to 8-week-old dairy calves administered a single dose (6.42 h) or adult dairy cattle administered multiple doses (5.20 h) of transdermal flunixin meglumine [14, 16]. However, plasma concentrations may not reflect pharmacodynamic effects. With NSAIDs, this may be due to binding within tissues or to the COX enzyme, as suspected with flunixin meglumine in dogs [22]. In African clawed frogs administered injectable flunixin meglumine, analgesic effects lasted for as long as 24 h following treatment, although plasma concentrations were not measured [3, 6]. Further study is warranted into the analgesic effects and duration of transdermal flunixin meglumine in amphibians.

The topical administration method used in the current study was easily accomplished and likely less painful for the animals than using an injectable technique. Topical administration was rapid and required a less extensive and shorter period of restraint than would be required for injection, making topical administration arguably less stressful. Although animals in the current study were briefly restrained to ensure consistency of drug administration, in a clinical setting, it would likely be possible to treat most animals without any manual restraint.

Consistent transdermal absorption for different drugs and among different species of amphibians should not be assumed. In one study, injectable ketoprofen or meloxicam was applied topically to smoky jungle frogs (*Leptodactylus pentadactylus*) resulting in remarkably different pharmacokinetic profiles. Ketoprofen was well absorbed, yielding plasma concentrations consistent with analgesia in other species, while meloxicam was undetectable [5]. Considering this example of inconsistent absorption for different injectable, nontransdermal formulations, expanding the use of drugs formulated for transdermal absorption may offer more effective topical therapeutics for amphibians.

In dogs, treatment with flunixin meglumine has been associated with gastrointestinal (GI) side effects and mucosal ulceration [23, 24]. More severe GI perforations have occurred in flunixin meglumine-treated dogs that had concurrent conditions, such as dehydration and/or GI parasites, or those that were treated concurrently with corticosteroids [22]. GI side effects have also been seen in equids treated with flunixin meglumine [25]. Renal pathology has been appreciated in multiple bird species treated with flunixin meglumine [26]. Despite these effects in other species, no clinical adverse effects or systemic histopathologic lesions were observed in the current study. A topical reaction was appreciated in only one of 12 toads examined, and that lesion was small, restricted to the superficial epidermis, and not clearly related to the flunixin meglumine. In cattle, mild topical reactions including damaged hairs, flaky and thickened skin were seen 3 to 7 days after dosing. The reactions were considered cosmetic and resolved without treatment [27]. In general, transdermal flunixin meglumine at 3.3 mg/kg appeared safe for marine toads, but animals should be monitored for development of dermal reactions. The effect of multiple doses of transdermal flunixin meglumine is unknown and warrants investigation.

There are limitations in pharmacokinetic studies, such as this one, where the number of available samples is restricted due to the animals' small size or intolerance for repeated handling. The technique of pooling samples has been used in similar studies [5], but while this is beneficial for small-sized animals where sample volume is limited, it ignores the variance among individuals. A population-based pharmacokinetic approach using sparse sampling is limited by not representing all animals at each sampling timepoint; however, sources of variability among each animal remain represented within the data. The sparse sampling method was effective in providing a safe method for pharmacokinetic investigation of a small species in the current study. It should be considered as an alternative to pooling samples if the animals' size allows a large enough sample for processing.

Housing these animals as a group may have provided a source of absorption variability. Following treatment, some toads were occasionally seen in physical contact with other toads. It is feasible that this may have allowed for additional drug absorption from one toad to another. In cattle, flunixin absorption was lower if animals were allowed access to other cows and licking of drug from the application site occurred. However, this did not prevent absorption of an effective dose and did not require dose adjustment [27]. Based on the data, there is no clear evidence group housing affected absorption in toads, either from individual outliers or evidence of additional absorption events. In clinical use, best practice may be to isolate animals for a period time immediately following treatment until there was no residue of the drug on the treated animal.

## 5. Conclusion

Based on the results of the current study, a single topical dose of transdermal flunixin meglumine at 3.3 mg/kg is rapidly absorbed in marine toads with minimal adverse effects. Comparisons to recent studies in domestic ruminants suggest the dose administered and plasma concentrations achieved in this study may be consistent with effective analgesia if marine toads follow a similar trend. The permeability of amphibian skin provides an advantageous route for topical therapy, especially in small species that are difficult to medicate by injectable or oral routes. Future studies should evaluate multiple dose pharmacokinetics and confirm safety, as well as analgesic effects of transdermal flunixin meglumine. A population-based pharmacokinetic approach using sparse sampling techniques can be useful for future amphibian pharmacokinetic studies, including those investigating transdermal therapeutics.

## Figures and Tables

**Table 1:** Pharmacokinetic parameters from a noncompartmental model using plasma data from toads (*n* = 21) given a single transdermal dose of 3.3 mg/kg flunixin meglumine. *λ*_z_ = rate constant associated with the terminal elimination phase; *t*_1/2*λ*z_ = half-life of the terminal elimination phase; AUC_0⟶∞_ = total area under the curve; %AUC_extrap_ = area under the curve extrapolated as a percentage of the total; CL/F = total body clearance; Vd = volume of distribution; *t*_max_ = time to maximum concentration; *C*_max_ = maximum plasma concentration.

Parameter	Units	Mean
*λ*_z_	h^−1^	0.25
*t*_1/2*λ*z_	h	2.79
AUC_0⟶_	h^∗^*µ*g/ml	29.37
%AUC_extrap_	%	0.28
CL/F	ml/h/kg	112.36
Vd/F	ml/kg	452.29
*t*_max_	h	1
*C*_max_	*µ*g/ml	6.31

## Data Availability

The full data set which was used for this research is available from the corresponding author upon request.

## References

[1] Terril-Robb L. A., Suckow M. A., Grigdesby C. F. (1996). Evaluation of the analgesic effects of butorphanol tartrate, xylazine hydrochloride, and flunixin meglumine in leopard frogs (*Rana pipiens*). *Contemporary Topics in Laboratory Animal Science*.

[2] Stevens C. W., MacIver D. N., Newman L. C. (2001). Testing and comparison of non-opioid analgesics in amphibians. *Journal of the American Association for Laboratory Animal Science*.

[3] Coble D. J., Taylor D. K., Mook D. M. (2011). Analgesic effects of meloxicam, morphine sulfate, flunixin meglumine, and xylazine hydrochloride in African-clawed frogs (*Xenopus laevis*). *Journal of the American Association for Laboratory Animal Science: JAALAS*.

[4] Minter L. J., Clarke E. O., Gjeltema J. L., Archibald K. E., Posner L. P., Lewbart G. A. (2011). Effects of intramuscular meloxicam administration on prostaglandin E2 synthesis in the North American bullfrog (*Rana catesbeiana*). *Journal of Zoo and Wildlife Medicine*.

[5] Balko J. A., Watson M. K., Papich M. G., Posner L. P., Chinnadurai S. K. (2018). Plasma concentrations of ketoprofen and meloxicam after subcutaneous and topical administration in the smoky jungle frog (*Leptodactylus pentadactylus*). *Journal of Herpetological Medicine and Surgery*.

[6] Smith B. D., Vail K. J., Carroll G. L. (2018). Comparison of etomidate, benzocaine, and MS222 anesthesia with and without subsequent flunixin meglumine analgesia in African clawed frogs (*Xenopus laevis*). *Journal of the American Association for Laboratory Animal Science*.

[7] Lees P., Reviere J. E., Papich M. G. (2018). Analgesic, anti-inflammatory, antipyretic drugs. *In Veterinary Pharmacology and Therapeutics*.

[8] Ardente A. J., Barlow B. M., Burns P., Goldman R., Baynes R. E. (2008). Vehicle effects on in vitro transdermal absorption of sevoflurane in the bullfrog, *Rana catesbeiana*. *Environmental Toxicology and Pharmacology*.

[9] Chinnadurai S. K., Kane L. P. (2014). Advances in amphibian clinical therapeutics. *Journal of Exotic Pet Medicine*.

[10] Riviere J. E., Shapiro D. P., Coppoc G. L. (1979). Percutaneous absorption of gentamicin by the leopard frog, *Rana pipiens*. *Journal of Veterinary Pharmacology and Therapeutics*.

[11] Mombarg M., Claessen H., Lambrechts L., Zwart P. (1992). Quantification of percutaneous absorption of metronidazole and levamisole in the fire-bellied toad (*Bombina orientalis*). *Journal of Veterinary Pharmacology and Therapeutics*.

[12] D’Agostino J. J., West G., Boothe D. M., Jayanna P. K., Snider T., Hoover J. P. (2007). Plasma pharmacokinetics of selamectin after a single topical administration in the American bullfrog (*Rana catesbeiana*). *Journal of Zoo and Wildlife Medicine*.

[13] Valitutto M. T., Raphael B. L., Calle P. P., Papich M. G. (2013). Tissue concentrations of enrofloxacin and its metabolite ciprofloxacin after a single topical dose in the coqui frog (*Eleutherodactylus coqui*). *Journal of Herpetological Medicine and Surgery*.

[14] Kleinhenz M. D., Van Engen N. K., Gorden P. J. (2016). The pharmacokinetics of transdermal flunixin meglumine in Holstein calves. *Journal of Veterinary Pharmacology and Therapeutics*.

[15] Thiry J., Fournier R., Roy O., Catala M. (2017). Evaluation of flunixin meglumine pour-on administration on prostaglandin E 2 concentration in inflammatory exudate after induction of inflammation in cattle. *Research in Veterinary Science*.

[16] Kleinhenz M. D., Gorden P. J., Smith J. S. (2018). Pharmacokinetics of multiple doses of transdermal flunixin meglumine in adult Holstein dairy cows. *Journal of Veterinary Pharmacology and Therapeutics*.

[17] Kleinhenz M. D., Gorden P. J., Smith J. S. (2019). Effects of transdermal flunixin meglumine on experimentally induced lameness in adult dairy cattle. *Journal of Dairy Science*.

[18] “Random Number Generator/Picker,” last modified 2020, https://andrew.hedges.name/experiments/random/

[19] Ette E. I., Williams P. J. (2004). Population pharmacokinetics I: background, concepts, and models. *Annals of Pharmacotherapy*.

[20] Reppert E. J., Kleinhenz M. D., Montgomery S. R. (2019). Pharmacokinetics and pharmacodynamics of intravenous and transdermal flunixin meglumine in meat goats. *Journal of Veterinary Pharmacology and Therapeutics*.

[21] Cramer M. C., Pairis‐Garcia M. D., Bowman A. S. (2019). Pharmacokinetics of transdermal flunixin in sows. *Journal of Veterinary Pharmacology and Therapeutics*.

[22] Vonderhaar M. A., Salisbury S. K. (1993). Gastroduodenal ulceration associated with flunixin meglumine administration in three dogs. *Journal of the American Veterinary Medical Association*.

[23] Dow S. W., Rosychuk R. A., McChesney A. E., Curtis C. R. (1990). Effects of flunixin and flunixin plus prednisone on the gastrointestinal tract of dogs. *American Journal of Veterinary Research*.

[24] Luna S. P. L., Basílio A. C., Steagall P. V. M. (2007). Evaluation of adverse effects of long-term oral administration of carprofen, etodolac, flunixin meglumine, ketoprofen, and meloxicam in dogs. *American Journal of Veterinary Research*.

[25] Mozaffari A., Derakhshanfar A., Alinejad A., Morovati M. (2010). A comparative study on the adverse effects of flunixin, ketoprofen and phenylbutazone in miniature donkeys: haematological, biochemical and pathological findings. *New Zealand Veterinary Journal*.

[26] Pereira M. E., Werther K. (2007). Evaluation of the renal effects of flunixin meglumine, ketoprofen and meloxicam in budgerigars (Melopsittacus undulatus). *Veterinary Record*.

[27] Banamine® Transdermal Technical Bulletin. Intervet Inc. 2018

